# Prevalence, Virulence, and Antimicrobial Resistance of Major Mastitis Pathogens Isolated from Taiwanese Dairy Farms

**DOI:** 10.3390/antibiotics13010036

**Published:** 2023-12-30

**Authors:** Bigya Dhital, Shih-Te Chuang, Jui-Chun Hsieh, Ming-Hsiu Hsieh, Hsin-I Chiang

**Affiliations:** 1 Department of Animal Science, National Chung Hsing University, Taichung 40227, Taiwan; d111037004@mail.nchu.edu.tw (B.D.); michelle89626@gmail.com (M.-H.H.); 2 Department of Veterinary Medicine, College of Veterinary Medicine, National Chung Hsing University, Taichung 40227, Taiwan; stchuang@dragon.nchu.edu.tw; 3 Department of Animal Science and Technology, National Taiwan University, Taipei 106319, Taiwan; jchsieh@ntu.edu.tw; 4 Smart Sustainable New Agriculture Research Center (SMARTer), Taichung 40227, Taiwan

**Keywords:** cows, mastitis, antimicrobial susceptibility, resistance, genotype, virulence gene

## Abstract

Mastitis, a highly prevalent disease in dairy cows, is responsible for massive financial losses due to decreased milk yield, milk quality, and costly medication. This research paper investigates antimicrobial susceptibility in cows and the role played by both resistance and virulence gene distribution in bovine mastitis. A total of 984 raw milk samples were collected from five different dairy farms and cultured on sheep blood agar plates. Antimicrobial susceptibility was determined by disc diffusion, and corresponding resistance and virulence genes were detected by PCR. Among the collected milk samples, 73, 32, and 19 isolates of *Streptococcus* spp., *Staphylococcus* spp., and coliforms were identified, respectively. The antimicrobial susceptibility results showed that *Streptococcus* spp. were resistant to tetracycline (86.30%), neomycin (79.45%), and oxacillin (73.97%). *Staphylococcus* spp. were resistant to tetracycline (59.37%) and oxacillin (53.12%). Lastly, coliforms were resistant to oxacillin (100%) and bacitracin (68.42%). The genotyping results showed that *Streptococcus* spp. carried the resistance genes *tetM* (46.57%) against tetracycline, *bcrB* (41.09%) against bacitracin, and *aph(3)-II* (39.72%) against neomycin. *Staphylococcus* spp. carried the resistance genes *bcrB* (40.62%) and *tetM* (18.75%), and coliforms carried the resistance genes *tetM* (42.10%) and *bcrB* (57.89%). Moreover, 57.53%, 75.0%, and 63.15% of *Streptococcus* spp., *Staphylococcus* spp., and coliforms carried *lmb, fib,* and *ompC* virulence genes, respectively. All three tested bacterial genera showed no significant association between antimicrobial resistance genes and virulence factors, although they were negatively correlated (*p* > 0.05). The combination of resistance gene identification and susceptibility tests as components of the diagnosis of bovine mastitis can help in selecting effective antimicrobial agents to treat it.

## 1. Introduction

Bovine mastitis is a common disease of dairy cattle worldwide that causes considerable economic losses due to decreased milk production, low milk quality, increased therapeutic costs, and early culling. Over 135 types of bacterial species have been recorded from bovine mastitis, but only 20 distinct pathogenic bacteria commonly cause mastitis in dairy animals [1,2]. The most common mastitis-causing etiological agents are bacteria, such as *Staphylococcus*, *Streptococcus*, *Escherichia coli*, mycoplasma, and other coliforms; other nonbacterial microorganisms, such as fungi and algae, can also cause bovine mastitis [3]. Antimicrobial agents are the main approach to treating and preventing bovine mastitis, and they have been considered the first choice against bacterial infection for a long time [4]. The frequent use of antimicrobial agents in food animals can result in the presence of antimicrobial-resistant bacteria in milk, meat, and other dairy products, which may pose food safety hazards to humans [5].

Antimicrobial resistance is a serious issue worldwide. Among the antimicrobial agents used on dairy farms, nearly 60–70% are used only for treating and preventing mastitis [4]. The selection pressure and overuse of antimicrobial agents in animal production might be the main reasons for the development of antimicrobial resistance in microorganisms and the emergence of multidrug-resistant bacteria, a serious threat to public health. Therefore, the World Health Organization has recommended the proper use of antimicrobial agents in the livestock industry [6]. Generally, bacteria evolve resistance through several mechanisms, including gene mutations, horizontal gene transfer, antimicrobial agent inactivation by enzymes, drug target modification, the alteration of membrane permeability, and efflux pumps [7,8]. Many antimicrobial resistance genes have been identified in bovine mastitis: *blaZ* and *blaTEM* (β-lactam resistance genes); *norA* (fluoroquinolone resistance gene); *tetM* and *tetK* (tetracycline resistance genes); and *ermA, ermB,* and *ermC* (erythromycin resistance genes) [9,10]. The study of gene mutation has become a standard tool to investigate antimicrobial resistance, which allows the investigation of the transmission of bacterial genetic material among host populations in more detail compared to conventional culture-based phenotypic resistance methods.

Bacteria possess several virulence factors that play important roles in pathogenesis in the causative microorganism. Resistance in bacteria may be related to the loss of virulence in different models of infections [11]. In one study, mice intraperitoneally injected with *E. coli* resistant to more than four types of antimicrobial agents showed higher survival rates than mice injected with the reference strain. Interestingly, *E. coli* bacteria susceptible to antimicrobial agents and resistant to two classes of antimicrobial agents showed lower survival rates in mice. This phenomenon is explained by the fitness cost of antimicrobial resistance [11].

In Taiwan, it is estimated that every year, approximately 70–76% of all antimicrobial agents are used to treat pets and farm animals [12]. Bovine mastitis is a severe constraint against Taiwanese livestock production because few antimicrobial agent susceptibility reports are available. Studying antimicrobial resistance in dairy cattle is crucial for the proper prevention and cure of bacterial infections. Therefore, the purpose of this study was to identify mastitis-causing bacteria and their antimicrobial resistance patterns and to investigate the association between antimicrobial resistance genes and virulence factors.

## 2. Results

### 2.1. Identification of Bacterial Isolates through 16S rRNA Gene Sequencing

Based on 16S rRNA gene sequencing, 73, 32, and 19 isolates were identified as *Streptococcus* spp., *Staphylococcus* spp., and coliforms, respectively (Table 1). Among the 73 isolates of *Streptococcus* spp., the predominant species identified was *Strep. uberis* (*n* = 30), followed by *Strep. lutetiensis* (*n* = 13) and *Strep. dysgalactiae* (*n* = 10). Similarly, among the 32 isolates of *Staphylococcus* spp., the predominant species was *Staph. aureus* (*n* = 14), followed by *Staph*. *epidermidis* (*n* = 7) and *Staph. hemolyticus* (*n* = 4). Furthermore, the dominant species among the 19 coliform isolates were *Escherichia coli* (*n* = 8), followed by *Enterobacter aerogenes* (*n* = 5) and *Klebsiella pneumoniae* (*n* = 4). 

### 2.2. Antimicrobial Susceptibility of Streptococcus spp., Staphylococcus spp., and Coliforms

The antimicrobial resistance status of *Streptococcus* spp., *Staphylococcus* spp., and coliforms are depicted in Table 2. Most *Streptococcus* spp. were resistant to tetracycline (86.30%), neomycin (79.45%), and oxacillin (73.97%). In contrast, *Streptococcus* spp. were susceptible to cephalothin (91.78%), cefuroxime (80.82%), and ceftiofur (73.97%). Among the tested *Staphylococcus* spp., 59.37% were resistant to tetracycline, followed by oxacillin (53.12%) and ampicillin (43.75%). However, all tested *Staphylococcus* spp. were susceptible to ceftiofur (100%), cephalothin (100%), and cefuroxime (100%). All tested coliforms bacteria were resistant to oxacillin (100%), and nearly 68% of isolates were resistant to bacitracin. However, coliforms were susceptible to ceftiofur (100%), cefuroxime (84.21%), and neomycin (78.94%)

### 2.3. Comparative Study of Phenotypic and Genotypic Antimicrobial Resistance in Streptococcus spp., Staphylococcus spp., and Coliforms

The results revealed a negative correlation between the phenotypic and genotypic antimicrobial resistance patterns of *Streptococcus* spp. for bacitracin (*p* < 0.0234), ampicillin (*p* < 0.0124), oxacillin (*p* < 0.0335), cefuroxime (*p* < 0.0059), and cephalothin (*p* < 0.0003); however, no significant associations were observed for tetracycline, neomycin, and ceftiofur, although the correlations were negative, as shown in Table 3. The phenotypic and genotypic antimicrobial resistance patterns of *Staphylococcus* spp. were negatively correlated with tetracycline (*p* < 0.0239), whereas no significant associations were observed for neomycin, bacitracin, ampicillin, and oxacillin, although they were negatively correlated (Table 4). Lastly, no significant associations were found between the phenotypic and genotypic antimicrobial resistance patterns of coliforms with tetracycline, bacitracin, and ampicillin (Table 5). 

### 2.4. Correlation between Antimicrobial Resistance Genes and the Virulence Factors of Bacterial Isolates

All three bacterial genera, *Streptococcus*, *Staphylococcus*, and coliforms, showed no association with antimicrobial resistance genes and virulence factors (Table 6), although they were negatively correlated.

### 2.5. Prevalence of Virulence Genes in Streptococcus spp., Staphylococcus spp., and Coliforms

**Figure 1:**
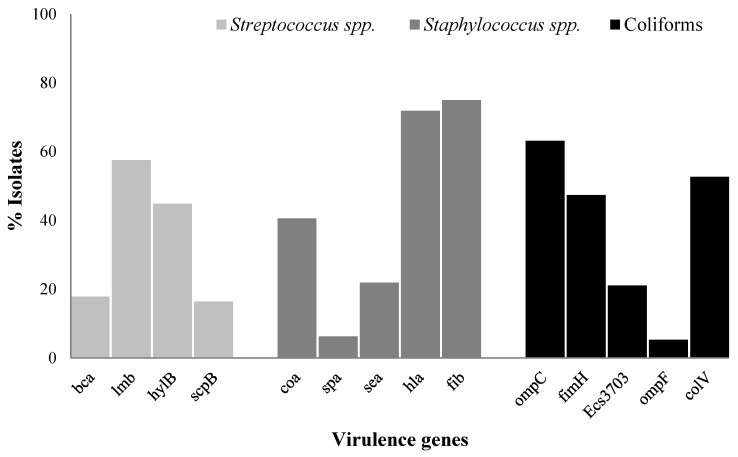
The prevalence of virulence genes in *Streptococcus* spp., *Staphylococcus* spp., and coliforms from bovine mastitis. *bca* = surface protein ą-C, *bac* = surface protein ß-C, *lmb* = laminin-binding protein, *hylB* = hyaluronidase, *scpB* = Streptococcal C5a peptidase, *coa* = coagulase, *spa* = protein A, *sea* = enterotoxin A, *hla* = alpha-hemolysin, and *fib* = fibrinogen-binding protein, *fimH* = type 1 fimbriae, *Ecs3703* = putative ABC transport protein, *ompC* and *ompF* = outer membrane protein, and *colV* = Colicin V.

The prevalence of the virulence genes in the tested bacterial genera is presented in Figure 1. A total of 57.53% of *Streptococcus* spp. carried the *lmb* virulence gene, followed by *hylB* (44.83%), *bca* (17.80%), and *scpB* (16.43%); however, none of them were found to be positive for the bac virulence gene. Similarly, 75.0% of *Staphylococcus* spp. carried the fib virulence gene, followed by *hla* (71.87%), *coa* (40.62%), *sea* (21.87%), and *spa* (6.25%). Likewise, 63.15% of coliforms harbored the *ompC* virulence gene, followed by *colV* (52.63%), *fimH* (47.36%), *Ecs3703* (21.05%), and *ompF* (5.26%). 

## 3. Discussion

Our study revealed that *Strep. uberis* was the most frequently identified bacterial species that caused bovine mastitis in Taiwan, whereas *Staph. aureus* and *E. coli* were also detected as major bacteria. Previously, *Strep. uberis* was detected as the most dominant mastitis-causing pathogen in Taiwan [13]. The primary source of *Strep. uberis* in dairy farms include bedding materials, water, soil, and plant matter; therefore, pathogens may easily enter animal udders and transmit between cows [14]. *Staph. aureus* has been widely recognized as a common pathogen in intramammary infections in dairy cows due to its high transmissibility and ability to cause chronic infections [15]. The present study identified certain coagulase-negative staphylococci (CoNS) species that cause mastitis in cattle. CoNS are emerging globally as opportunistic pathogens, and their infections are usually self-limiting; however, studies have reported the need for antimicrobial treatment in clinical mastitis cases [16]. Similarly, *E. coli* is one of the major pathogens that causes bovine mastitis [11]. This study revealed that *Klebsiella pneumoniae* and *Enterobacter aerogenes* also caused bovine mastitis, as with other previous findings [17,18]. Therefore, numerous factors may determine bacterial presence in dairy farms, such as environment, management systems, temperature, humidity, and barn design [19].

Determining antimicrobial susceptibility profiles is essential for effective therapy and monitoring the selection and emergence of antimicrobial-resistant microorganisms. Our study found that most *Streptococcus* spp. were resistant to tetracycline and neomycin, which is similar to previous findings in Taiwan [13] and Northwest China [20]. Tetracycline is the most widely used antibiotic globally to treat various infections in cattle, including Taiwan, due to its broad-spectrum effectiveness, which may be the reason for the widespread resistance against tetracycline. The efflux pump is the most important mechanism of bacterial antimicrobial resistance to tetracycline, although ribosomal protective protein and enzyme inactivation also have a role in resistance development [21]. Aminoglycosides (neomycin) are used for prophylactic purposes in dairy animals, but they are not an effective antimicrobial agent for the treatment of mastitis-causing *Streptococcus* bacteria because most streptococci have inherited resistance to this class of antimicrobial due to their poor ability to penetrate the cell walls of bacteria [22]. Most of the tested *Staphylococcus* spp. and coliforms were susceptible to neomycin on our studied farms. The results also showed that 61.64% of *Streptococcus* spp. were susceptible to bacitracin, which is a much higher percentage than previous findings [23], but lower than the findings of [13]. These differing results might be due to differences in sampling areas or different antimicrobial agent usage histories. The present study’s isolates showed higher susceptibility to cephalothin, cefuroxime, and ceftiofur. This might be due to less exposure in these dairy farm environments, different antimicrobial agents being rotated in the treatment of dairy animals, or the broad-spectrum nature of these antimicrobials. Nearly half of the tested *Streptococcus* spp. (45.20%) and *Staphylococcus* spp. (43.75%) and one-third of coliforms (31.57%) developed resistance to ampicillin, as observed in previous findings on *Staphylococcus* and coliforms [22,24], while contrasting with the findings of [13] regarding *Streptococcus*. Ampicillin intramammary ointment is highly accessible for mastitis treatments in Taiwan; therefore, bacteria are likely to develop resistance against ampicillin on dairy farms.

In the present study, a high proportion of resistance genes related to tetracycline (*tetM*), neomycin (*aph(3)-II*), bacitracin (*bcrB*), and β-lactam (*blaZ*) were observed in *Streptococcus* spp., *Staphylococcus* spp., as well as coliforms, which have great potential to lead to high resistance rates against these antimicrobial agents. The present study revealed that 15.62% of *Staphylococcus* spp. carried the *mecA* gene, but one study found that nearly 2% of *Staph. aureus* carried the *mecA* gene [25]. The presence of the beta-lactam resistance gene *blaZ* varied depending on bacterial type. The present study demonstrated that 79.45% of *Streptococcus* spp. were resistant to neomycin based on the results of the phenotypical assay, but only 20.54% of *Streptococcus* spp. carried the resistance gene (*aph (3)- I*). Very few coliforms showed resistance to tetracycline (31.57%) and neomycin (21.05%) in terms of phenotypic assay, whereas much higher numbers of coliforms harbored the corresponding tetracycline (68.42%) and neomycin (57.89%) resistance genes. Our findings suggest that phenotypic resistance does not necessarily rely on the existence of resistance genes. In the present study, *Streptococcus* spp. showed no association between the phenotypic and genotypic antimicrobial resistance patterns for some antimicrobial agents, such as tetracycline, neomycin, and ceftiofur. Similarly, a previous study also mentioned that the majority of *Streptococcus* spp. showed no association between the phenotypic and genotypic characteristics of some antimicrobial agents [26]. These results could be explained by various reasons. Firstly, the majority of bacteria in this study showed a positive resistance phenotype but a negative genotype, which might be explained by the limited number of antimicrobial resistance genes investigated. Therefore, it was necessary for all possible antimicrobial resistance genes to be examined. Secondly, resistance gene expression depends on the existence of a promoter or inducer. Resistance genes distant from a promoter or associated with a weak promoter may lead to hindered gene expression. Thirdly, resistance genes might remain unexpressed due to point mutations [27]. Lastly, the small sample sizes and low number of observations could also contribute to these findings. *Staphylococcus* spp. showed a negative correlation between phenotypic and genotypic antimicrobial resistance to tetracycline; however, a previous study found a positive correlation in *Staph. aureus* [24]. 

In general, increased resistance is linked directly or indirectly to decreased virulence and fitness [28]. This is because developing resistance is a genetic burden and associated with a fitness cost [11,27]. Murine models have shown that penicillin-susceptible *Strep. pneumoniae* was virulent, although some isolates with low penicillin susceptibility were nonvirulent [29]. Similarly, *E. coli* resistant to tigecycline showed significantly decreased virulence in a mouse model [30]. In the present study, all three tested bacterial genera showed no association between antimicrobial resistance genes and virulence factors. Perhaps the sample size in the current study was insufficient or under antimicrobial resistance conditions. When bacteria are under the selective pressure of antimicrobials, it weakens the association between antimicrobial resistance and bacterial virulence [31]. Fitness costs in antimicrobial-resistant bacteria should be further studied to elucidate the underlying evolutionary mechanisms for resistance genes’ emergence, stability, and dissemination.

Multiple virulence factors play significant roles in host cell adhesion, invasion, and evasion of the host immune response. Our current findings on virulence factor distribution align with previous studies [32,33,34] but contradict the results reported by [11,35]. Virulence factors have diverse roles in bacterial pathogenesis. For example, laminin-binding protein (*lmb*) is vital in facilitating adherence to host laminin [36], and fibrinogen-binding protein (*fib*) is a major plasma protein that is crucial in blood clotting, inflammation, and interactions with cells and the extracellular matrix [37]. Additionally, both the *ompF* and *ompC* genes encode major porin proteins that act as passive diffusion channels for nutrients, antimicrobial agents, and small molecules [38]. Hence, these virulence factors might be crucial in mastitis development or persistence.

## 4. Conclusions

*Streptococcus* spp. was more dominant than *Staphylococcus* spp. and coliforms in causing bovine mastitis in Taiwan. These three bacterial genera revealed high-level phenotypic resistance to certain antimicrobial agents. The presence of bacterial resistance and diverse virulence profiles among these pathogens is concerning. Effective antimicrobials, cefuroxime, cephalothin, and ceftiofur, were identified for pathogen treatment, but their usage must be carefully monitored to prevent resistance development. The lack of associations between antimicrobial resistance genes and virulence factors in the tested bacterial genera may be influenced by factors such as bacterial species, host immunity, virulence mechanisms, and environmental conditions. Combining resistance gene identification and susceptibility tests can aid farmers in selecting appropriate chemotherapeutic measures. Regular monitoring of mastitis-causing pathogens is vital to assess antimicrobial resistance patterns in dairy herds.

## 5. Materials and Methods

### 5.1. Herd Enrollment Criteria and Milk Sample Collection

A total of 984 raw milk samples were collected from five different commercial dairy farms in Taiwan. Farm details are presented in Figure S1. Farms were required to have at least 200 lactating cows. Herds must participate in regular Dairy Herd Improvement testing or the monthly California Mastitis Test (CMT) must be used for all lactating cows with a yearly farm survey by sending individual cow milk samples to a reference laboratory. Quarter milk samples (including clinical, subclinical, and suspicious mastitis samples) were collected monthly from the one dairy farm in Tainan. For the other four farms, only quarter milk samples from cows diagnosed with clinical mastitis were collected. Veterinarians examined clinical mastitis samples, characterized by observable changes in milk or systemic symptoms. Subclinical mastitis samples, representing intramammary infections lacking clinical symptoms, were identified using the CMT. Raw milk samples were collected using aseptic procedures as described by the National Mastitis Council (https://www.nmconline.org/nmc-protocols-guidelines-and-procedures/) (accessed on 13 March 2020).

### 5.2. Isolation and Identification of Bacteria

Milk samples (10 µL) were cultured on 5% sheep blood agar and incubated at 37 °C for 20–24 h (Creative Biotechnology Company, New Taipei City, Taiwan). Bacteria were identified based on colonial characteristics and microscopic examination. Although different mastitis-causing bacterial genera were identified, only *Staphylococcus* spp., *Streptococcus* spp., *Escherichia coli*, and other coliforms were selected for further study because they are more common on dairy farms. Bacteria were preserved in tryptic soy broth (Becton, Dickinson and Company, Taipei City) with 20% glycerol (BIONOVAS biotechnology Co., Ltd., Toronto, Canada) and stored at −80 °C for further study.

### 5.3. Bacterial Species Identification through 16S rRNA Gene Sequencing

Bacterial DNA was extracted using the PureLinkTM Microbiome DNA Purification Kit’s recommended protocol (Invitrogen, Thermo Fisher Scientific, USA, Waltham, MA), and DNA was quantified using a MicroDrop (BIO-DL). Samples were amplified by PCR using 16S rRNA gene targeting primers [39]. PCR was performed on a T100 Thermal Cycler (Bio-Rad) with primers, and PCR conditions are summarized in the Supplementary Materials (Table S1). PCR amplicons were analyzed via electrophoresis in 1.5% agarose gels (*w*/*v*) and visualized using a Gel Doc XR+ System. A single discrete PCR amplicon band (458 bp) was purified using a QIAquick PCR Purification kit (QIAGEN, Inc., Toronto, ON, Canada). Purified PCR products were sent for sequencing with forward and reverse primers at National Chung Hsing University biotechnology center in Taichung, Taiwan. The sequencing data were analyzed using the National Center for Biotechnology Information rRNA/ITS nucleotide database. 

### 5.4. Antimicrobial Susceptibility Test

Antimicrobial susceptibility tests were performed using the Kirby–Bauer disc diffusion method on Mueller–Hinton (M-H) agar plates. Eight commercially prepared antimicrobial discs were used: ampicillin (10 μg), oxacillin (1 μg), cephalothin (30 μg), cefuroxime (30 μg), ceftiofur (30 μg), neomycin (30 μg), bacitracin (10 units), and tetracycline (30 μg). Bacterial inoculum (5 × 10^5^ cfu/mL) was inoculated on M-H agar plates according to the Clinical and Laboratory Standards Institute (CLSI) recommendations, and antimicrobial discs were placed and incubated at 37 °C for 20–24 h. The responses of the isolates to various antimicrobial agents were evaluated by measuring the zone of inhibition diameter and interpreting results according to standards recommended by [40], and bacitracin results were interpreted based on [41]’s recommendation. *Staphylococcus aureus* subsp. *aureus Rosenbach* ATCC 25923 was used as the quality control strain.

### 5.5. Identification of Antimicrobial Resistance Genes

Bacterial DNA was used as the template for PCR amplification. All isolates were tested through the PCR amplification of genes that confer resistance to neomycin (*aph (3)-I*; *aph(3)-II*), β-lactam (*mecA*, *blaZ,* and *ampC*), bacitracin (*bcrA* and *bcrB*), tetracycline (*tetM, tetO, tetA,* and *tetB*), and 16S Nossa were used as positive controls, and a PCR mix without a DNA template was used as negative control in all assays. Primers and PCR conditions used in this study are listed in Supplementary Materials (Table S1). Amplicons were analyzed via electrophoresis, as stated before. 

### 5.6. Identification of Virulence Genes

Fifteen virulence genes were selected for *Staphylococcus* spp. (*coa, spa, sea, hla*, and *fib*), *Streptococcus* spp. (*bac, bca, lmb, hylB,* and *scpB*), and coliforms (*ompC, fimH, Ecs3703, ompF,* and *colV*). Primers and PCR conditions used in this study are listed in Supplementary Materials (Table S2). Amplicons were analyzed via electrophoresis, as stated before.

### 5.7. Statistical Analysis

Pearson correlation coefficient values were calculated using SAS 9.4 (SAS Institute Inc., Cary, NC, USA) to determine associations between the phenotypic and genotypic resistance patterns of antimicrobial agents and the relationship between antimicrobial resistance genes and bacterial virulence factors. *p* ≤ 0.05 was considered statistically significant.

## Figures and Tables

**Table 1:** Identification of bacterial isolates through 16S rRNA gene sequencing.

Genus	Species	Number (*n*)	Accession Number
*Streptococcus* (*n* = 73)	*Strep. Uberis*	30	NR_040820
	*Strep. Lutetiensis*	13	NR_037096
	*Strep. Dysgalactiae*	10	NR_027517
	*Strep. Bovis*	9	AJ305257
	*Strep. Equinus*	6	NR_042052
	*Strep. Agalactiae*	5	OP752129
*Staphylococcus* (*n* = 32)	*Staph. Aureus*	14	NR_037007
	*Staph. Epidermidis*	7	NR_036904
	*Staph. Hemolyticus*	4	AY688062
	*Staph. Chromogenes*	3	AY688044
	*Staph. Hyicus*	2	NR_036905
	*Staph. Simulans*	1	AY688101
	*Staph. Capitis*	1	NR_027519
Coliforms (*n* = 19)	*Escherichia coli*	8	X80721
	*Enterobacter aerogenes*	5	LT221165
	*Klebsiella pneumoniae*	4	NR_036794
	*Escherichia fergusonii*	2	NR_027549

**Table 2:** Antimicrobial susceptibility test results for *Streptococcus* spp., *Staphylococcus* spp., and coliforms.

Organism	Antimicrobial Susceptibility ^a^	TET	NEO	BAC	AMP	OXA	CXM	CF	XNL
*Streptococcus* (*n* = 73)	Resistant (%)	86.30	79.45	38.35	45.20	73.97	19.17	8.21	26.02
	Susceptible (%)	13.69	20.54	61.64	54.79	26.02	80.82	91.78	73.97
*Staphylococcus* (*n* = 32)	Resistant (%)	59.37	21.87	34.37	43.75	53.12	0.00	0.00	0.00
	Susceptible (%)	40.62	78.12	65.62	56.25	46.87	100	100	100
Coliforms (*n* = 19)	Resistant (%)	31.57	21.05	68.42	31.57	100	15.78	31.57	0.00
	Susceptible (%)	68.42	78.94	31.57	68.42	0.00	84.21	68.42	100

^a^ All intermediately resistant isolates are considered susceptible. TET = tetracycline, NEO = neomycin, BAC = bacitracin, AMP = ampicillin, OXA = oxacillin, CXM = cefuroxime, CF = cephalothin, and XNL = ceftiofur.

**Table 3:** Comparative study of phenotypic and genotypic antimicrobial resistance in *Streptococcus* spp.

Antimicrobial Agents	Gene(s)	Characteristics of *Streptococcus* Isolates ^1^					Association	
		P^+^/G^+^ (*n*)	P^−^/G^-^ (*n*)	P^+^/G^−^ (*n*)	P^−^/G^+^ (*n*)	G^+^ (%)	r ^2^	*p* ^3^
Tetracycline	Total	42	3	21	7	67.12	−0.21822	0.0696
	*tetM*	31			3	46.57		
	*tetB*	5			0	6.84		
	*tetA*	3			2	6.84		
	*tetO*	3			2	6.84		
Neomycin	Total	39	10	19	5	60.27	−0.19294	0.1298
	*aph(3)-I*	13			2	20.54		
	*aph(3)-II*	26			3	39.72		
Bacitracin	Total	22	24	6	21	58.90	−0.32350	0.0234 *
	*bcrB*	16			14	41.09		
	*bcrA*	6			7	17.80		
Ampicillin	Total	20	29	13	11	42.46	−0.37388	0.0124 *
	*blaZ*	12			9	28.76		
	*ampC*	8			2	13.69		
Oxacillin	Total	35	10	19	9	60.27	−0.26827	0.0335 *
	*blaZ*	25			6	42.46		
	*ampC*	10			3	17.80		
Cefuroxime	Total	9	42	5	17	35.61	−0.48324	0.0059 *
	*blaZ*	6			16	30.13		
	*ampC*	3			1	5.47		
Cephalothin	Total	2	55	4	12	19.17	−0.75593	0.0003 *
	*blaZ*	2			12	19.17		
	*ampC*	0			0	0.00		
Ceftiofur	Total	11	50	8	4	20.54	−0.33508	0.1181
	*blaZ*	7			2	12.32		
	*ampC*	4			2	8.21		

^1^ P^+^, phenotypic resistance; P^−^, phenotypic susceptibility; G^+^, resistance-gene-positive; G^−^, resistance-gene-negative. ^2^ Association between resistant phenotypes and resistance genes. ^3^ *p* ≤ 0.05 was considered significant, and the significant values are represented by *.

**Table 4:** Comparative study of phenotypic and genotypic antimicrobial resistance in *Staphylococcus* spp.

Antimicrobial Agents	Gene(s)	Characteristics of *Staphylococcus* Isolates ^1^					Association	
		P^+^/G^+^ (*n*)	P^−^/G^−^ (*n*)	P^+^/G^−^ (*n*)	P^−^/G^+^ (*n*)	G^+^ (%)	r ^2^	*p* ^3^
Tetracycline	Total	10	6	9	7	53.12	−0.44164	0.0239 *
	*tetM*	5			1	18.75		
	*tetB*	0			2	6.25		
	*tetO*	3			3	18.75		
	*tetA*	2			1	9.37		
Neomycin	Total	5	17	2	8	40.62	−0.41931	0.1197
	*aph(3)-I*	2			3	15.62		
	*aph(3)-II*	3			5	25.00		
Bacitracin	Total	10	13	1	8	56.25	−0.20101	0.4093
	*bcrB*	7			6	40.62		
	*bcrA*	3			2	15.62		
Ampicillin	Total	9	13	5	5	43.75	−0.35714	0.1333
	*blaZ*	5			3	25.00		
	*ampC*	1			0	3.12		
	*mecA*	3			2	15.62		
Oxacillin	Total	7	13	10	2	28.12	−0.36155	0.1283
	*blaZ*	4			2	18.75		
	*ampC*	1			0	3.12		
	*mecA*	2			0	6.25		
Cefuroxime	Total	0	31	0	1	3.12	NT	NT
	*blaZ*	0			1	3.12		
	*ampC*	0			0	0.00		
	*mecA*	0			0	0.00		
Cephalothin	Total	0	31	0	1	3.12	NT	NT
	*blaZ*	0			1	3.12		
	*ampC*	0			0	0.00		
	*mecA*	0			0	0.00		
Ceftiofur	Total	0	31	0	1	3.12	NT	NT
	*blaZ*	0			1	3.12		
	*ampC*	0			0	0.00		
	*mecA*	0			0	0.00		

^1^ P^+^, phenotypic resistance; P^−^, phenotypic susceptibility; G^+^, resistance-gene-positive; G^−^, resistance-gene-negative. ^2^ Association between resistant phenotypes and resistance genes. ^3^ *p* ≤ 0.05 was considered significant, and the significant values are represented by *. NT, correlation coefficients (r value) cannot be calculated (at least one variable is constant).

**Table 5:** Comparative study of phenotypic and genotypic antimicrobial resistance in coliforms.

Antimicrobial Agents	Gene(s)	Characteristics of Coliforms Isolates ^1^					Association	
		P^+^/G^+^ (*n*)	P^-^/G^-^ (*n*)	P^+^/G^-^ (*n*)	P^-^/G^+^ (*n*)	G^+^ (%)	r ^2^	*p* ^3^
Tetracycline	Total	5	5	1	8	68.42	−0.32026	0.2643
	*tetM*	4			4	42.10		
	*tetO*	1			0	5.26		
	*tetB*	0			2	10.52		
	*tetA*	0			2	10.52		
Neomycin	Total	4	8	0	7	57.89	NT	NT
	*aph(3)-I*	1			3	21.05		
	*aph(3)-II*	3			4	36.84		
Bacitracin	Total	9	4	4	2	57.89	−0.23652	0.3960
	*bcrB*	9			2	57.89		
	*bcrA*	0			0	0.00		
Ampicillin	Total	4	8	2	5	47.36	−0.43033	0.1864
	*blaZ*	4			5	47.36		
	*ampC*	0			0	0.00		
Oxacillin	Total	7	0	12	0	36.84	NT	NT
	*blaZ*	6			0	31.57		
	*ampC*	1			0	5.26		
Cefuroxime	Total	2	16	1	0	10.52	NT	NT
	*blaZ*	2			0	10.52		
	*ampC*	0			0	0.00		
Cephalothin	Total	2	12	4	0	10.52	NT	NT
	*blaZ*	1			0	5.26		
	*ampC*	1			0	5.26		
Ceftiofur	Total	0	19	0	0	0.00	NT	NT
	*blaZ*	0			0	0.00		
	*ampC*	0			0	0.00		

^1^ P^+^, phenotypic resistance; P^−^, phenotypic susceptibility; G^+^, resistance-gene-positive; G^−^, resistance-gene-negative. ^2^ Association between resistant phenotypes and resistance genes. ^3^ *p* ≤ 0.05 was considered significant. NT, correlation coefficients (r value) cannot be calculated (at least one variable is constant).

**Table 6:** The correlation between antimicrobial resistance genes and virulence factors of bacteria.

Bacteria Species	r ^1^	*p* ^2^
*Streptococcus* (*n* = 73)	−0.04193	0.7247
*Staphylococcus* (*n* = 32)	−0.22953	0.2063
Coliforms (*n* = 19)	−0.01996	0.9354

^1^ Association between antimicrobial resistance genes and virulence factors. ^2^ *p* ≤ 0.05 was considered significant.

## Data Availability

Data are contained within the article.

## Supplementary Materials

The following supporting information can be downloaded at: https://www.mdpi.com/article/10.3390/antibiotics13010036/s1, Figure S1: Milk sample collection sites in Taiwan during 2020–2021. Table S1: Oligonucleotide sequences, primers, and targets for Polymerase Chain Reaction amplification of antimicrobial resistance genes. Table S2: Oligonucleotide sequences, primer names, and conditions of Polymerase Chain Reaction amplification for target virulence genes.
