# Supplementary material for: Prevalence, Virulence, and Antimicrobial Resistance of Major Mastitis Pathogens Isolated from Taiwanese Dairy Farms
Source: Antibiotics (Basel). 2023 Dec 30;13(1):36. doi: 10.3390/antibiotics13010036 (PMC10812822; doi:10.3390/antibiotics13010036)
Supplement: Supplementary file 1 [file antibiotics-13-00036-s001.zip › antibiotics-2768426-supplementary.pdf]

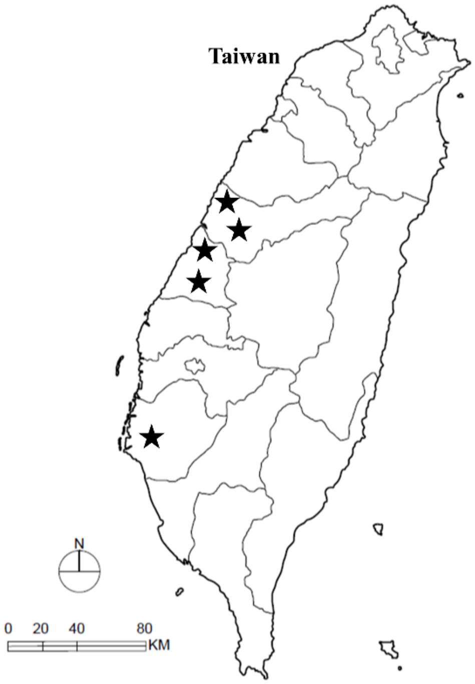

Figure S1: Milk samples collection sites in Taiwan during 2020-2021.

Two farms in Taichung (24.4040 N, 120.6482 E and 24.5263 N, 120.7486E, respectively), two farms in Chunghwa, (24.0288 N, 120.3624 E and 24.1376 N, 120.6666 E, respectively), and one farm in Tainan. (23.3327 N, 120.3340 E).

Table S1. Oligonucleotide sequences, primers, and targets for Polymerase Chain Reaction amplification of antimicrobial resistance genes

Antibiotics class	Genes	Primers	Oligonucleotide sequence (5′→3′)	Amplified product (bp)	Annealing temperature (°C)	References
(Aminoglycoside)	*aph*	*aph (3)-I*-F	ATGGGCTCGCGATAATGTC	600	52	Maynard *et al.* (2003)
Neomycin	*(3)-I*	*aph (3)-I*-R	CTCACCGAGGCAGTTCCAT			
	aph	*aph (3)-II*-F	ATGGGCTCGCGATAATGTC	680	54	Maynard *et al.* (2003)
	(3)-II	*aph (3)-II*-R	CTCACCGAGGCAGTTCCAT			
(Beta-lactamase)	*mecA*	*mecA*-1	AAAATCGATGGTAAAGGTTGGC	533	55	Frey *et al.* (2013)
Oxacillin		*mecA*-2	AGTTCTGCAGTACCGGATTTGC			
Ampicillin	*blaZ*	*blaZ*-F	CAGTTCACATGCCAAAGAG	772	48	Schnellmann *et al.*
Ceftiofur		*blaZ*-R	TACACTCTTGGCGGTTTC			(2006)
Cephalothin	*ampC*	*ampC*-F *ampC*-R	ATGATGAAAAAATCGTTATGC	1,143	53	Winokur *et al.* (2001)
Cefuroxime			TTGCAGCTTTTCAAGAATGCGC			
Bacitracin	*bcrA*	*bcrA*-F	CCGCAATGAAAATGATGTTG	584	49	Matos *et al.* (2009)
		bcrA-R	TGCGGCTATCTTACCATCTG			
	bcrB	*bcrB*-F	AAAGAAACCGACTGCTGATA	489	48	Matos *et al.* (2009)
		bcrB-R	GCTTACTTGTATAGCAGAGA			
Tetracycline	*tetM*	*tetM*-F	AGGGCATCAAGCAACATTTC	366	49	Pyatov *et al.* (2017)
		tetM-R	TCGAGGTCCGTCTGAACTTT			
	tetO	*tetO*-F	TAGCGGAACATTGCATTTGA	290	48	Pyatov *et al.* (2017)
		tetO-R	TTTCTGTAAGTGCCCCAAGC			
	tetA	*tetA*-F	TGTCCGACAAGTTGCATGAT	178	49	Pyatov *et al.* (2017)
		tetA-R	CCTTGAACGGCCTCAATTT			
	tetB	*tetB*-F	CTCCTTGGCTTGGAAAAATG	229	50	Pyatov *et al.* (2017)
		tetB-R	AACCAACCGAACCACTTCAC			
	16S	Nossa F	GGAGGCAGCAGTRRGGAAT	458	58	Nossa *et al.* (2010)
	rRNA	Nossa R	CTACCRGGGTATCTAATCC			

Table S2. Oligonucleotide sequences, primer names, and conditions of Polymerase Chain Reaction amplification for target virulence genes

Genes	Primers	Oligonucleotide sequence (5′→3′)	Amplified product (bp)	Annealing temperature (°C)	References
		Staphylococcus spp.,			
coa	*coa*-F	ATAGAGATGCTGGTACAGG	544	48	Kalorey *et al.* (2007)
	coa-R	GCTTCCGATTGTTCGATGC			
spa	*spa*-F	CAAGCACCAAAAGAGGAA	487	46	Kalorey *et al.* (2007)
	spa-R	CACCAGGTTTAACGACAT			
sea	*sea*-F	GCAGGGAACAGCTTTAGGC	521	53	Løvseth *et al.* (2004)
	sea-R	GTTCTGTAGAAGTATGAAACACG			
hla	*hla*-F	GGTTTAGCCTGGCCTTC	550	49	Booth *et al.* (2001)
	hla-R	CATCACGAACTCGTTCG			
fib	*fib*-F	CTACAACTACAATTGCCGTCAACAG	404	56	Tristan *et al.* (2003)
	fib-R	GCTCTTGTAAGACCATTTTCTTCAC			
		Streptococcus spp.,			
bac	*bac*-F	TGTAAAGGACGATAGTGTGAAGAC	530	54	Dmitriev *et al.* (2002)
	bac-R	CATTTGTGATTCCCTTTTGC			
bca	*bca*-F	TAACAGTTATGATACTTCACAGAC	535	56	Dmitriev *et al.* (2002)
	bca-R	ACGACTTTCTTCCGTCCACTTAGG			
lmb	*lmb*-F	ACCGTCTGAAATGATGTGG	572	48	Dmitriev *et al.* (2002)
	lmb-R	GATTGACGTTGTCTTCTGC			
hylB	*hylB*-F	ACAAATGGAACGACGTGACTAT	346	51	Dmitriev *et al.* (2002)
	hylB-R	CACCAATTGGCAGAGCCT			
scpB	*scpB*-F	CCAAGACTTCAGCCACAAGG	591	53	Dmitriev *et al.* (2002)
	scpB-R	CAATTCCAGCCAATAGCAGC			
		coliforms			
ompC	*ompC*-F	TTAGAACTGGTAAACCAGACCCA	1,104	54	Zhang *et al.* (2018)
	ompC-R	ATGAAAGTTAAAGTACTGTCCCTCC			
fimH	*fimH*-F	TGCAGAACGGATAAGCCGTGG	508	57	Bicalho *et al.* (2010)
	fimH-R	GCAGTCACCTGCCCTCCGGTA			
Ecs3703	*Ecs3703*-F	TTGACATCATCAATCACCAATG	693	49	Zhang *et al.* (2018)
	Ecs3703-R	TCAATGTTGGACCGAATGTG			
ompF	*ompF*-F	TTTCCAAGGGTAACGGTGAA	382	50	Zhang *et al.* (2018)
	ompF-R	CCATCAACCAGACCAAAGAAG			
colV	*colV*-F	GGAAATGACCTGAATGCTGG	399	52	Zhang *et al.* (2018)
	colV-R	CCGCTCATGCATCAGTACC			
